# Suturing under tension in minimally invasive surgery: A comparison of three intracorporeal knot types: Philipp Romero_1_, Hans Kessler_1_, Juri Fuchs_1,_ Estelle Willuth_1_, Frank Pianka_2_, Patrick Günther_1_

**DOI:** 10.1007/s00423-025-03829-y

**Published:** 2025-08-06

**Authors:** Philipp Romero, Hans Kessler, Juri Fuchs, Estelle Willuth, Frank Pianka, Patrick Günther

**Affiliations:** 1 Division of Paediatric Surgery, Department of General, Visceral and Transplantation Surgery, University Hospital Heidelberg, Im Neuenheimer Feld 430, D-69120 Heidelberg, Germany; 2 Department of General, Visceral and Transplantation Surgery, University Hospital Heidelberg, Im Neuenheimer Feld 420, 69120 Heidelberg, Germany

**Keywords:** Intracorporeal knot tying, Minimally invasive surgery, Surgical square knot, Slipping knot

## Abstract

**Purpose:**

Intracorporeal knot tying (ICKT) and suturing under tension are essential skills for performing advanced minimally invasive surgery (MIS) procedures. Over recent years, various intracorporeal knot types with specific properties have been developed. The classical surgical square knot (CSK) continues to be used as the standard knot in MIS. Previously published studies suggested that the security of the surgical square knot improves when the knot throw combination is adjusted from four wraps (2W1W1W) to five wraps (3W1W1W). Additionally, the intracorporeal slipping knot (SLK) has been found to be superior to the CSK in sutures under tension. This study aimed to compare ICKT of the CSK, a modified square knot (MSK) and the SLK during simulated suture placement under tension.

**Methods:**

A laparoscopic box trainer and a standardized silicone suture pad was utilized for ICKT. The participants consisted of medical students, surgical residents and senior physicians. A standardized video demonstrating the ICKT variants was shown to all participants before evaluation. The procedural implementation, knot quality, total task time and accuracy were evaluated.

**Results:**

A total of 267 knots were included in the study. Compared to the CSK, the MSK showed significantly better results across the entire group in terms of time, knot quality, and procedural implementation score.

**Conclusion:**

In the evaluation of all participants’ knots, the MSK showed significantly better results for all variables except accuracy compared to the CSK. The MSK showed similarly good results to the SLK. Both the MSK and SLK appear particularly well-suited for tension sutures.

## Introduction

Intracorporeal knot tying (ICKT) and suturing are essential skills for performing advanced minimally invasive surgery (MIS) procedures like Nissen fundoplication. Particularly suture placement under tension presents a notable challenge in MIS. Over recent years, various intracorporeal knot types with specific properties and training approaches have been developed [1, 2]. Licensed MIS training programs such as those of FLS (The Fundamentals of Laparoscopic Surgery) in the USA or AGE (Arbeitsgemeinschaft Gynäkologische Endoskopie, Working Group for Gynecological Endoscopy) in Germany teach and evaluate the intracorporal surgical square knot [3]. The classical surgical square knot consists of an initial double-wrap throw (2 W) followed by two single-wrap (1 W) throws in opposing directions (2W1W1W) and is likewise used most frequently in MIS [4]. But the surgical square knot can sometimes loosen with minimal force, making it potentially unsafe [5]. With a intracorporeal suture under tension, the challenge of tying a secure knot increases further [1]. In a prospective study with dynamometric measurement, a significant increase in security was shown by simply changing the knot throwing sequence of the square knot to 3W1W1W [5]. Other studies have shown that, during intracorporeal suturing under tension, the slipping knot exhibits superior quality compared to the surgical square knot [1, 4]. Which knot variant is best suited for a suture under tension? And how effectively can these different knot sequences be learned and applied? This study aimed to compare ICKT of the classical surgical square knot (CSK), the modified square knot (MSK) and the slipping knot (SLK) during simulated suture placement under tension across three groups with varying education levels.

## Methods

This monocentric prospective study was performed in the MIS training center of the Department of General, Visceral, and Transplantation Surgery at Heidelberg University. A laparoscopic box trainer (KARL STORZ GmbH, Tuttlingen, Germany) with predefined openings was utilized for ICKT. Two working ports were inserted through these openings at 30° angles on either side of a centrally positioned 10-mm-diameter laparoscope with 30° optics (KARL STORZ GmbH). Two lockable laparoscopic needle holders (KARL STORZ GmbH) were placed in the lateral ports. The laparoscope was connected to a camera box (Telecam Pal; KARL STORZ GmbH) and a Xenon 300 light source (KARL STORZ GmbH). The image was displayed on a 14-inch monitor (Sony Trinitron, Tokyo, Japan) positioned at a fixed height directly in front of the proband. A 12-cm suture (Ethibond 3/0, Excel, RB 1, 17 mm, 1/2c; Ethicon, Johnson & Johnson, Somerville, NJ) was positioned centrally. A standardized silicone suture pad with a 0.5 cm wound dehiscence and marked needle entry and exit points adjacent to the incision was secured to the base of the box trainer. The silicone used had a hardness grade of 5 on the Shore-A scale. All types of knots were assessed across three groups with varying levels of surgical experience. The first group comprised 16 medical students (5 female, 11 male) in their third to sixth year of a six-year program in Germany, with no prior laparoscopic surgery experience; clinical training begins in the third year. The second group (*n* = 22) consisted of 13 female and 9 male surgical residents in their first to fourth years of residency, who had previously performed over 10 laparoscopic procedures but fewer than 20 laparoscopic sutures. The third group (*n* = 5) included one female and four male senior physicians, each with experience in at least 100 laparoscopic procedures and over 20 laparoscopic sutures. All participants registered voluntarily for the study. All participants in group 1 and 2 received a one-hour hands-on training session from the same instructor on tying each ICKT variant. The variant was switched every 20 min, beginning with CSK, followed by SLK, and then MSK. A 1:1 student-to-instructor ratio was maintained throughout the training. The three intracorporeal knot variants were performed using the C-loop technique. The fundamental ICKT procedures have been described in detail in previous publications [1, 2, 4]. A standardized video demonstrating the ICKT variants was shown to all participants before evaluation. Medical students and residents tied two of each knot variant immediately after their hands-on training session. Senior physicians tied four of each knot variant. A single observer evaluated all assessment parameters for each participant in real time. The validated point-by-point scoring system chosen for this study enabled a near-objective, real-time assessment of the variables [1]. The procedural implementation was evaluated using a modified 23-point checklist originally described by Munz et al. [6]. Knot quality was measured on a quantifiable five-point scale, as outlined by Muresan et al. [7], which assesses quality based on tightness (1 point), holding ability (2 points), sufficient knot throwing sequence (1 point) and alignment of the incision edges on the suture pad (1 point). Two points were awarded if the knot maintained tissue approximation under tension. To assess this, the silicone suture pad was removed from its fixation and manual tension was applied via a pulley mechanism. The applied force approximated the level of tension typically used in clinical practice, such as during the testing of intestinal anastomoses or skin sutures. Total task time (in seconds) was recorded from the moment the needle was loaded onto the needle holder until the suture was placed and secured with the final throw. Accuracy was measured as the average deviation (in mm) of the thread’s entry and exit points from the designated markings on the suture pad. Proficiency for intracorporeal CSK and SLK using the C-loop technique was defined as meeting the following criteria: attaining at least 18 out of 23 points on the procedural implementation checklist, achieving a score of 4 or higher out of 5 on the knot quality scale, and completing the task within 120 s [1]. The dropout criteria included the knots torn out of the silicone pad during ICKT. Statistics for all variables were calculated using SPSS version 26.0 (IBM Corp., Armonk, USA). The empirical distribution of continuous data was described using means and standard deviations. Intergroup differences between variables were assessed using the independent samples t test. Intragroup comparisons were made using the dependent samples t test. The level of significance in this study was *P* <.05.

All procedures involving human participants were in accordance with the ethical standards of the institutional and national research committee and with the 1964 Helsinki Declaration and its later amendments. This study was approved by the local ethics committee at Heidelberg University (S-334/2011 and S-436/2018).

## Results

A total of 267 knots were included in the study. In the student group, a total of 83 knots were evaluated, with 13 dropouts. Among the residents, no dropouts were recorded (*n* = 132), while the senior physicians had 8 dropouts (*n* = 52). Compared to the CSK, the MSK showed significantly better results across the entire group in terms of time, knot quality, and procedural implementation score (Table 1). In the analysis of all participants, no significant differences were found between the MSK and SLK (Table 1). The senior physicians showed significantly better results than the medical students in terms of time and procedure for all knot types (Table 2). However, the students performed significantly better in knot quality for the CSK compared to senior physicians (Table 2). For the CSK and MSK, the senior physicians were significantly superior to the residents only in terms of time and procedural implementation score (Table 3). Regarding achieving the competency level for procedure time, only senior physicians were able to record more than 25% of all knots. While students consistently achieved values of > 40% at the competency level for knot quality, the other two groups showed a heterogeneous pattern, with less than 15% of the CSK above the threshold. The execution of knot procedures and accuracy showed high values of > 85% across all groups and knot types.

**Table 1:** Comparison of node types in all subjects

	CSK (n=87)	MSK (*n*=91)	*p*	MSK (*n*=91)	SLN (*n*=89)	*p*
Task time (sec±SD)	193.5±72.6	171±49.9	0.01	171±49.9	183.3±44.2	n.s.
Knot Quality (score±SD)	2.7±1.3	3.4±1.3	0.001	3.4±1.3	3.4±1.3	n.s.
Accuracy (mm±SD)	0.1±0.3	0.1±0.3	n.s.	0.1±0.3	0.1±0.3	n.s.
Procedural implementation (score±SD)	20.2±3.2	21.2±1.3	0.008	21.2±1.3	21.2±1.3	n.s.

*CSK* classical surgical square knot, *MSK* modified square knot, *SLK* slipping knot, *n.s.* not significant

**Table 2:** Comparison of students and senior physicians at ICKT

	Students			Senior physicians			Senior physicians vs Students		
	CSK (*n*=27)	MSK (*n*=28)	SLN (*n*=28)	CSK (*n*=16)	MSK (*n*=19)	SLN (*n*=17)	CSK (*p*)	MSK (*p*)	SLN (*p*)
Task time (sec±SD)	216.3±72.1	186.9±57.1	191.2±38.7	150.3±61	141.2±38.4	163.5±47.6	0.004	0.004	0.03
Knot Quality (score±SD)	3.3±1.5	3.5±1.3	3.5±1.3	2.3±1	3.7±1.2	3.6±1.2	0.03	n.s.	n.s.
Accuracy (mm±SD)	0.3±0.4	0.3±0.4	0.3±0.4	0.1±0.3	0.1±0.3	0.1±0.2	n.s.	n.s.	n.s.
Procedural implementation (score±SD)	20.1±1.7	21.0±1.3	21.1±1.1	21.8±1.6	22.2±0.8	21.8±1	0.002	0.002	0.03

*CSK* classical surgical square knot, *MSK* modified square knot, *SLK* slipping knot, *n.s.* not significant

**Table 3:** Comparison of residents and senior physicians at ICKT

	Residents			Senior Physicians			Senior Physicians vs Residents		
	CSK (*n*=44)	MSK (*n*=44)	SLN (*n*=44)	CSK (*n*=16)	MSK (*n*=19)	SLN (*n*=17)	CSK (*p*)	MSK (*p*)	SLN (*p*)
Task time (sec±SD)	195.3±71	173.8±44.5	185.9±44.8	150.3±61	141.2±38.4	163.5±47.6	0.02	0.007	n.s.
Knot Quality (score±SD)	2.6±1.2	3.3±1.4	3.3±1.3	2.3±1	3.7±1.2	3.6±1.2	n.s.	n.s.	n.s.
Accuracy (mm±SD)	0.1±0.3	0.1±0.2	0.1±0.3	0.1±0.3	0.1±0.3	0.1±0.2	n.s.	n.s.	n.s.
Procedural implementation (score±SD)	19.7±4.1	20.9±1.3	21.1±1.5	21.8±1.6	22.2±0.8	21.8±1	0.05	0.000	n.s.

*CSK* classical surgical square knot, MSK modified square knot, SLK slipping knot, n.s. not significant

## Discussion

ICKT under tension presents a challenge in minimally invasive surgery [1, 4]. There is a wide variety of knot types, techniques, and teaching methods available in training facilities for minimally invasive surgery [1, 2, 4, 8]. However, the question of the optimal knot type for sutures under tension has not been definitively answered. In this monocentric prospective study conducted at the MIS Training Center of Heidelberg University, three types of intracorporeal knots were compared: the square knot in both its classical (CSK) and modified forms (MSK), and the SLK. The CSK continues to be used as the standard knot in minimally invasive surgery and is taught in certified training programs such as the FLS [3]. A previously published study using a dynamometric measurement method demonstrated significantly better quality outcomes when the square knot was adjusted from 4 (2W1W1W) to a total of 5 wraps (3W1W1W) or throws [5]. In the study by Romeo et al., the CSK opened in 3.8% of cases at a force of less than 1 N [N] [5]. The 3W1W1W combination of 5 throws never opened below 30 N [5]. The authors acknowledge that a purely tensiometric measurement of knot security using forces exceeding 30 N, as reported by Romeo et al., holds limited clinical relevance in MIS [5]. For this reason, our study employed a validated 5-point scoring system [7] to assess knot quality, which incorporates simulated wound edge approximation under applied tension. Additional clinically relevant evaluation parameters such as knotting time and precision of entry and exit points were also included in the analysis. Our primary focus was on the practical implementation of the knot types and the associated challenges experienced by participants with varying levels of training. The SLK was deliberately chosen as the reference knot, as a previous study using the same experimental setup showed that the SLK outperformed the CSK in sutures under tension [4]. In the evaluation of all participants’ knots, the MSK showed significantly better results for all variables except accuracy compared to the CSK (Table 1). In addition to significantly better knot quality, the MSK did not exhibit any disadvantages in terms of time or procedural implementation despite requiring an additional throw. On the contrary, securing the knot with three initial throws more frequently led to the adaptation of simulated wound edges and reduced the need for corrections during the procedure. This resulted in significantly higher scores in procedural implementation and knot quality, and faster knot completion overall. The MSK showed comparable results to the SLK across all variables (Table 1). Although no statistical significance was observed, the MSK could even be tied faster than the SLK. In the square knot procedure, the intermediate step of pulling the knot throw into the sliding position and guiding it to the base, which is crucial in the SLK, is eliminated [4]. This allows the procedure to be completed more quickly overall. The SLK has proven particularly effective in tissues under tension during suturing—such as in thoracoscopic diaphragmatic hernia repairs, hiatoplasties, or fundoplication [9]. Once the second throw is flipped into the sliding position, the knot can be tightened in a controlled manner, locked in place, and used to approximate tissue over a considerable distance. This sliding mechanism also facilitates suturing in anatomically challenging regions—for example, in pelvic reconstructive procedures such as sacrocolpopexy or post-hysterectomy repairs [10]. We have used the SLK, among other indications, for diaphragmatic plication in cases of phrenic nerve injury following cardiac surgery in children. In this thoracoscopically assisted procedure, particularly in infants under one year of age, the working space is small, visibility is limited due to the ventilated lung, and the procedure duration must be minimized due to limited tolerance for CO₂ insufflation or anesthesia [11]. In contrast to the SLK, fewer clinical studies are currently available on the MSK. Deveau et al. highlight the advantages of the initial 3-throw sequence (3-1-1) compared to the CSK (2-1-1) [12]. In the 3-1-1 technique, the initial loops prevent slippage of the suture, allowing optimal tension to be maintained through the subsequent throws. Each throw adds to the stability of the knot, ultimately resulting in a strong, durable configuration that resists both breakage and unravelling [12]. Interestingly, our study found no significant difficulties in performing three consecutive throws compared to one or two, across all groups (see procedural implementation scores for SLK > 20, Tables 2 and 3). One drawback of the MSK, however, is its relatively larger knot size, which can make burial of the knot more challenging [12]. In contrast, the SLK offers the advantage of controlled regrasping of the leading, tensioned suture strand without compromising the knot’s tightness. With square knots, this is more difficult, as both suture ends must be pulled simultaneously to lock the knot. This characteristic may be particularly advantageous in confined operative spaces. Overall, the findings of our study, together with existing clinical data, suggest that both knot types may be applied in similar surgical scenarios. Future clinical studies could investigate whether the time efficiency of the MSK compared to the SLK can be confirmed, and whether the sliding feature of the SLK offers measurable advantages over the MSK in small or anatomically restricted spaces. In group comparisons with students, senior physicians demonstrated significantly better results in terms of time and procedural execution for all knots (Table 1). This expected outcome aligns with findings from comparable studies [1, 4, 13]. With increasing experience in ICKT, improvements are particularly notable in procedure time, as well as in the quality and safety of execution [1, 4]. Surprisingly, students were the only group to achieve average quality scores of > 3 for the classical square knot (CSK) (Tables 2 and 3). A primary focus emphasized during the preliminary hands-on training was knot quality, and the students effectively adhered to this priority, investing more time to improve knot quality (Tables 2 and 3). A standardized one-hour training session not only significantly improves students’ technical skills [4] but also directs their focus toward the specific demands of the knot technique being practiced. In contrast, for the experienced surgeons in our study, execution of the knot types was expected solely after viewing an instructional reference video.

Practicing surgeons typically develop individualized work habits through years of clinical experience. These include an emphasis on efficiency and speed [14] and the practical decision to avoid applying a method perceived as suboptimal — for instance, the use of a CSK under high-tension suture conditions. Surgeons are specifically trained to identify and apply alternative solutions when a chosen method proves ineffective [15]. Moreover, frustration in response to failure may be more pronounced when individuals perceive a high expectation of competence in the given technique. However, when individuals with high external performance expectations experience early setbacks, they are more likely to grapple with concerns about managing others’ perceptions and the risk of embarrassment. Consequently, under challenging conditions, they may be less inclined to persist compared to those with lower external expectations [16]. The difference bet ween residents and senior physicians was only significant for the CSK and MSK in terms of procedural execution and procedure time (Table 3). The more experience surgeons have in MIS, the easier they can implement or modify new procedures like the MSK. The fact that residents were able to approximate the results of senior physicians at the SLK could be explained by the fact that the SLK is used as the standard node in the MIC at Heidelberg University Hospital and residents are most familiar with. Except for the knot quality of the CSK (residents 2.6 ± 1.2, senior physicians 2.3 ± 1, Table 3), the other results for the CSK and SLK were similar to comparable studies [1]. In a previous MIS study using the CSK during a simulated tension suture in a similar setting, residents achieved an average knot quality score of 4.1, while senior physicians scored 3.5 [4]. A possible explanation for the differing results in the current study could be the adjustment of the silicone mixture of the suture pad from a hardness level of 0 Shore [4] to 5 Shore. The adaptation to the harder tissue, accompanied by greater tensile strength, seems particularly challenging. The effect that even a minimal deviation in movement results in an insufficient knot was amplified.

The establishment of a competency level for an intracorporeal knot-tying procedure in MIS is treated heterogeneously in the literature, and no uniform standards exist [1, 4, 8, 17]. In the Fundamentals of Laparoscopic Surgery (FLS) test, a CSK is expected to be completed within 10 min for a suture without tension [3]. Nickel et al. reported an average time of 75 s for a CSK on a suture without tension [8], while Romero et al. recorded 120 s [18]. These figures are aligned with the timing for open CSK knots, which are typically completed in on average of 30 s by surgeons [17]. Time is a particularly critical factor in MIS, especially when compared to open surgical procedures. Numerous studies have shown that the number of postoperative complications—such as nausea, thromboembolisms, infections, core hypothermia, and cardiopulmonary insufficiency—increases with longer operative durations [19, 20]. Besides the burden on the patient, operative time also carries health policy and economic significance, with costs ranging from $22 to $135 per operative minute [14]. In terms of achieving the competency level for procedure time in the current study, only senior physicians managed to complete more than 25% of all knots (Table 4). This result highlights how challenging ICKT is and underscores the need for MIS training programs at all levels of education. MIS training on the endotrainer particularly improves ICKT speed [1, 2, 8, 17, 18]. The heterogeneous results in this study regarding achieving the competency level for knot quality are related to the use of different knot types, the participants’ experience, and their prioritization during the procedure. However, a clear trend emerged among residents and senior physicians: compared to the CSK, the competency level was achieved more often with the MSK and SLK (Table 4). Residents were able to achieve a quality score of *≥* 4 out of 5 points for approximately every third knot with the MSK and SLK, while senior physicians achieved this for about every second knot. In addition to the level of training, the experimental setup with a simulated suture under tension on the silicone pad also affects the results. The standardized suture pad highlights the strengths and weaknesses of the participants and knot-tying techniques but only partially reflects the tissue conditions in vivo. When it comes to knot quality, no compromises should be made in vivo. Every suture must hold sufficiently. When a suture fails, the consequences may be disastrous, with possibly massive bleeding, evisceration, and wound dehiscence [5].

**Table 4:** Display of the ICKT competency level of all participants

	Students			Residents			Senior physicians		
	CSK (*n*=27)	MSK (*n*=28)	SLN (*n*=28)	CSK (*n*=44)	MSK (*n*=44)	SLN (*n*=44)	CSK (*n*=16)	MSK (*n*=19)	SLN (*n*=17)
Task time (<120 sec), n (%)	/	2 (7.1%)	/	4 (9.1%)	2 (4.5%)	1 (2.3%)	4 (25%)	5 (26,3%)	5 (29.4%)
Knot Quality (> 4), n (%)	13 (48.1%)	12 (42.8%)	12 (42.8%)	6 (13.6%)	16 (36,4%)	15 (34%)	1 (6.25%)	9 (47.4%)	7 (41.1%)
Accuracy (<2mm), n (%)	27 (100%)	28 (100%)	28 (100%)	44 (100%)	44 (100%)	44 (100%)	16 (100%)	19 (100%)	17 (100%)
Procedural implementation (>18), n (%)	25 (92.6%)	28 (100%)	28 (100%)	38 (86.4%)	43 (97.7%)	44 (100%)	16 (100%)	19 (100%)	17 (100%)

*CSK* classical surgical square knot, *MSK* modified square knot, *SLK* slipping knot

## Limitations

Our study had several limitations. No randomization was performed regarding the training or evaluation sequence of the knot techniques. Given the strictly limited training time of one hour, we deliberately started with the simplest knot (CSK) and progressively increased the complexity from SLK to MSK. This approach was intended to minimize frustration during training while maintaining consistency across groups for statistical comparison. However, a potential training or concentration bias in the comparison of knot types cannot be ruled out. For future studies with larger sample sizes, the use of a randomization protocol or a crossover learning model could enhance the statistical robustness. Current methods for assessing knot quality, including validated scoring systems, rely in part on subjective evaluation. Integrating sensor-based data collection and advanced analytical approaches, such as machine learning, may enable more objective and reproducible performance assessments in future research [21]. A single observer assessed all evaluation parameters for each participant in real time. The absence of a second (blinded) rater limited the objectivity of the findings and precluded calculation of interrater reliability. Nevertheless, the results clearly demonstrate the respective advantages of the different ICKT techniques. The validated point-by-point scoring system selected for this study enabled a near-objective, real-time assessment of the variables, thereby reducing personnel and time resources. We believe that our setup is well suited for performance assessment in surgical training.

## Conclusion

In our study, conducted in a simulated clinical setting, we were able to confirm the findings of Romeo et al. [5], which demonstrated qualitative advantages of MSK over CSK. The MSK was comparable to other standard intracorporeal knots, such as the SLK, in a simulated tension suture setting. The MSK showed similarly good results to the SLK. Both the MSK and SLK appear particularly well-suited for tension sutures compared to the CSK. Implementing a sufficient intracorporeal CSK in a suture under tension requires more time and a particular focus on knot quality. Dynamometric measurements of knots and validated scores for knot quality in a simulated wound under tension ultimately only provide indications of the advantages of different intracorporeal knot types. Clinical outcomes from patient application are needed to confirm the simulation-related data in real-world settings.

## Data Availability

No datasets were generated or analysed during the current study.
